# Hierarchical Plant Responses and Diversity Loss after Nitrogen Addition: Testing Three Functionally-Based Hypotheses in the Inner Mongolia Grassland

**DOI:** 10.1371/journal.pone.0020078

**Published:** 2011-05-19

**Authors:** Qingmin Pan, Yongfei Bai, Jianguo Wu, Xingguo Han

**Affiliations:** 1 State Key Laboratory of Vegetation and Environmental Change, Institute of Botany, Chinese Academy of Sciences, Beijing, China; 2 School of Life Sciences and Global Institute of Sustainability, Arizona State University, Tempe, Arizona, United States of America; 3 Sino-US Center for Conservation, Energy, and Sustainability (SUCCESS), Inner Mongolia University, Hohhot, China; Umea University, Sweden

## Abstract

**Background:**

Numerous studies have shown that nitrogen (N) deposition decreases biodiversity in terrestrial ecosystems. To explain the N-induced species loss, three functionally based hypotheses have been proposed: the aboveground competition hypothesis, the belowground competition hypothesis, and the total competition hypothesis. However, none of them is supported sufficiently by field experiments. A main challenge to testing these hypotheses is to ascertain the role of shoot and root competition in controlling plant responses to N enrichment. Simultaneously examining both aboveground and belowground responses in natural ecosystems is logistically complex, and has rarely been done.

**Methodology/Principal Findings:**

In a two-year N addition experiment conducted in a natural grassland ecosystem, we investigated both above- and belowground responses of plants at the individual, species, and community levels. Plants differed significantly in their responses to N addition across the different organizational levels. The community-level species loss was mainly due to the loss of perennial grasses and forbs, while the relative abundance of plant species was dependent mainly on individual-level responses. Plasticity in biomass allocation was much smaller within a species than between species, providing a biological basis for explaining the functionally based species loss. All species increased biomass allocation to aboveground parts, but species with high belowground allocations were replaced by those with high aboveground allocations, indicating that the increased aboveground competition was the key process responsible for the observed diversity loss after N addition in this grassland ecosystem.

**Conclusions/Significance:**

Our findings shed new light on the validity of the three competing hypotheses concerning species loss in response to N enrichment. They also have important implications for predicting the future impacts of N deposition on the structure and functioning of terrestrial ecosystems. In addition, we have developed a new technique for ascertaining the roles of aboveground and belowground competition in determining plant responses to N fertilization.

## Introduction

The global level of nitrogen (N) deposition has risen significantly since the industrial revolution, and is predicted to increase by 50∼100% from 2000 to 2030 [1], [2]. N enrichment is widely considered a major threat to plant species diversity in terrestrial ecosystems [3]–[7]. Understanding the mechanisms of biodiversity loss due to N deposition is important for unraveling the relationship between biodiversity and ecosystem functioning, and urgently needed for ecosystem management and environmental policy making.

**Figure 1:**
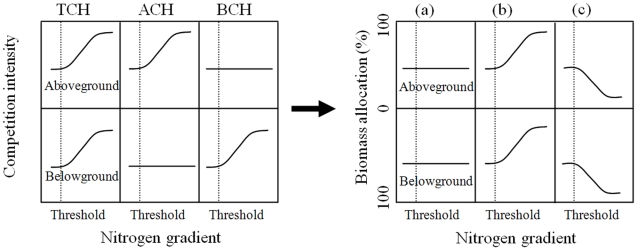
Three functionally-based hypotheses on diversity loss after nitrogen addition (left panel): total competition hypothesis (TCH), aboveground competition hypothesis (ACH), and belowground competition hypothesis (BCH), and their predictions of biomass allocation responses (right panel): (a) predictions from TCH, (b) predictions from ACH, and (c) predictions from BCH.

Several hypotheses on the mechanisms of plant diversity declining with N enrichment have been proposed, which focus either on random processes or competitive processes of species. The random loss hypothesis stresses the role of random deaths of small individuals of all species after N enrichment, leading to community-level thinning and the extinction of rare species [8]–[10]. In contrast, the functionally-based hypotheses emphasize the differences between species in terms of plant functional traits that determine their competitive abilities. Three functionally-based hypotheses have attracted most attention (Fig. 1). The aboveground competition hypothesis (ACH) suggests that a shift from belowground competition to aboveground competition after N enrichment results in the loss of poor aboveground competitors [11], [12]. In contrast, the belowground competition hypothesis (BCH) suggests that N fertilization creates resource patches which can be pre-empted by species with developed root systems, consequently leading to the loss of poor belowground competitors [13]. The total competition hypothesis (TCH) argues that the diversity loss due to N enrichment results from the enhanced intensity of both aboveground and belowground competition, with the superior competitors excluding the inferior ones [14]. Each of these hypotheses has been supported by some experiments but refuted by others, suggesting that the mechanisms underling N-induced biodiversity loss are complicated and system dependent.

Resource allocation plays a central role in regulating plant activities, such as growth, development, reproduction, and defense [15]–[17]. Because terrestrial plants face an unavoidable dilemma of resource allocation to aboveground versus belowground parts due to the physical separation of essential resources (e.g. light versus soil nutrients), from an evolutionary point of view, the allocation pattern between shoots and roots must be shaped by natural selection, and thus represent the ecological strategies of plant species to adapt to the constraints of their environment [18]. The interspecific differences in allocation pattern, to a great extent, determine the relative dominance of species in a plant community [19]. It is well documented that species dominant in nutrient-poor habitats usually have higher belowground allocation whereas species dominant in nutrient-rich habitats generally have higher aboveground allocation [20]. However, plants may alter their allocation patterns under changing environmental conditions [21]–[23]. Olff (1992) found that plant species exhibited high plasticity in biomass allocation under different combinations of light and nutrient supply when they each were grown alone in pots [24]. Both interspecific differences and intraspecific plasticity in allocation pattern may influence the outcome of species competition following N enrichment. If the intraspecific plasticity is larger than the interspecific difference, plant diversity loss cannot be attributed to the evolutionary divergence in allocation pattern between species. If the opposite is true, then evolutionary allocation patterns play an important role in influencing species diversity. However, the relative effects of evolutionary allocation patterns and allocation plasticity on species diversity loss have never been examined directly through a field experiment in a natural community.

Therefore, this study was designed to fill this gap by simultaneously comparing the roles of between-species differences and within-species plasticity in biomass allocation, measured by coefficients of variation (CVs), through a N addition experiment in the Inner Mongolia grassland in northern China. Elsewhere we already reported that N addition led to a significant decrease in plant species richness in this natural grassland ecosystem [25]. This study focused on the examination of the underlying mechanisms. Because species richness significantly increased for rare species (annuals) but decreased for dominant species (perennials) [25], which contradicts the prediction of the random loss hypothesis, we only examined the three functionally-based hypotheses. Specifically, we tested the following predictions based on three functionally-based hypotheses (Fig. 1). First, if total competition is the primary mechanism, the strength of both aboveground and belowground competition will increase after N addition. Co-occurring species should increase their individual growth without changing their allocation patterns (Fig. 1a). Species with smaller individuals will be excluded by those with larger individuals, and changes in species relative abundance (V_RAB_) should be correlated with individual biomass rather than allocation patterns. Second, if aboveground competition is the primary mechanism, N addition should facilitate the growth of species with higher aboveground allocation, leading to an increase in aboveground allocation responses (Fig. 1b). Consequently, V_RAB_ will be positively correlated with species allocation patterns or allocation responses of aboveground parts. Third, if belowground competition is the primary mechanism, N addition should facilitate the growth of species with higher belowground allocation, leading to an increase in belowground allocation responses (Fig. 1c). In this case, V_RAB_ should be positively correlated with belowground allocation patterns or allocation responses.

## Methods

### Experimental site

This experiment was conducted in an mature steppe ecosystem in Inner Mongolia Autonomous Region, China (E 116°42′, N 43°38′, elevation 1250 m a.s.l.) with the permit of Inner Mongolia Grassland Ecosystem Research Station (IMGERS),Chinese Academy of Sciences. This site (500 m×500 m) has been fenced to exclude large grazing animals since 1979 for long-term vegetation monitoring and experimental studies. The average annual temperature is 0.3°C, ranging from −21.6°C in January to 19.0°C in July, and the average annual precipitation is 346.1 mm, falling mainly during the growing season between May and August. The soil is dark chestnut [26], which is slightly alkaline (pH = 7.8). No fertilizer had been used prior to this experiment. The plant community is composed of 51 species from 28 families, and dominated by a perennial rhizome grass (*Leymus chinensis*) and a perennial bunch grass (*Stipa grandis*) [25]. The plant cover is about 30–40%, with about 11–18 species in a 1-m^2^ quadrat.

### Experimental design

The experiment was established in 1999, which included 7 treatments in total, each with 9 replicates (5 m×5 m plots). The 63 plots were arranged following a randomized block design, and separated from each other by 1 m buffers. Six levels of N addition (0, 1.75, 5.25, 10.5, 17.5, and 28.0 g N.m^−2^.yr^−1^) were created by adding NH_4_NO_3_ to plots early July (July 1–5) from 2000. To ensure that N was the only limiting element, following nutrients and trace elements were added: P (10 g P_2_O_5_·m^−2^·yr^−1^), S (0.2 mg·m^−2^·yr^−1^), Zn (190 µg·m^−2^·yr^−1^), Mn (160 µg·^m−2^·yr^−1^), and B (31 µg·m^−2^·yr^−1^). We also had a set of control plots without any nutrient addition.

### Target species and sampling

We selected 10 species for measuring biomass, abundance, and allocation patterns (Table S1). These species were present in each plot and together constituted 85–90% of the aboveground biomass and 89–90% of vegetation cover of the plant community. They also represent a broad spectrum of families, functional groups, and species ranks of the study ecosystem. Plant individuals (both above- and belowground parts) of target species were harvested using two steel frames (0.4×0.4×0.4 m) that were inserted into soil 0.4 m in depth. Sampling was conducted during August 28–31 2001, corresponding to peak biomass of most species.

For 5 grasses and 1 sedge species, we sampled 3–5 individuals of each species in each plot, to assure that 30 individuals were obtained for each treatment. For 2 annuals and 2 semi-shrubs that were scarce in abundance, we sampled 1–3 individuals for each species in each plot to obtain 15 individuals for each treatment. Soil cores were taken within the steel frame and washed gently with tap water to get roots. The roots were then placed into containers filled with deionized water. The shoots and roots of target species were identified and separated based on their aboveground parts. Plant materials were oven dried at 65°C for 48 h and then weighted accurate to 10^−4^ g. For the bunch grasses (*S. grandis*, *Cleistogenes squarrosa*, *Agropyron cristatum*, and *Achnatherum sibiricum*) and the sedge species (*Carex korshinskyi*), a bunch was counted as an individual. For the rhizomatous grass (*L. chinensis*), a tiller was treated as an individual. For other species, individual biomass was measured by shoot. In addition to biomass, the density of each plant species was measured with a 1×1 m quadrat in each plot.

### Data analysis

At the level of individual plants, we measured the individual biomass (IB), the belowground biomass (BB), and the aboveground biomass (AB). We calculated biomass allocation as follows: Belowground allocation (BA) = BB/IB; Aboveground allocation (AA) = AB/IB. We also computed the coefficients of variations (CVs) in IB, BA and AA as indices of plasticity for these variables. We estimated the biomass response of individuals as: R_IB_ = ln (IBt/IBc), where IBt and IBc are the mean biomass of individuals in the treatment and control plots, respectively. A positive R_IB_ value indicates an increase in individual biomass for a given species after N addition. In the same way, we estimated the belowground allocation response (R_BA_) and aboveground allocation response (R_AA_).

At the species level, we calculated the relative abundance of plant species as: RAB = plant density of a species/plant density of the community. Change in species relative abundance was estimated as: V_RAB_ = ln (RABt/RABc), where RABt and RABc are the mean relative abundance of a given species in treatment and control plots, respectively. Similarly, we calculated the species richness change at the community level.

Considering that the measurements of variables for co-occurring species in the same plot may not be completely independent, we used linear mixed models to test the overall effects of species, N addition rate, and their interaction. For each species, we also carried out separate ANOVAs to examine the effects of N addition rates. Duncan's multiple range tests at a significance level of 0.05 were used for multiple comparisons.

## Results

### Species relative abundance

**Figure 2:**
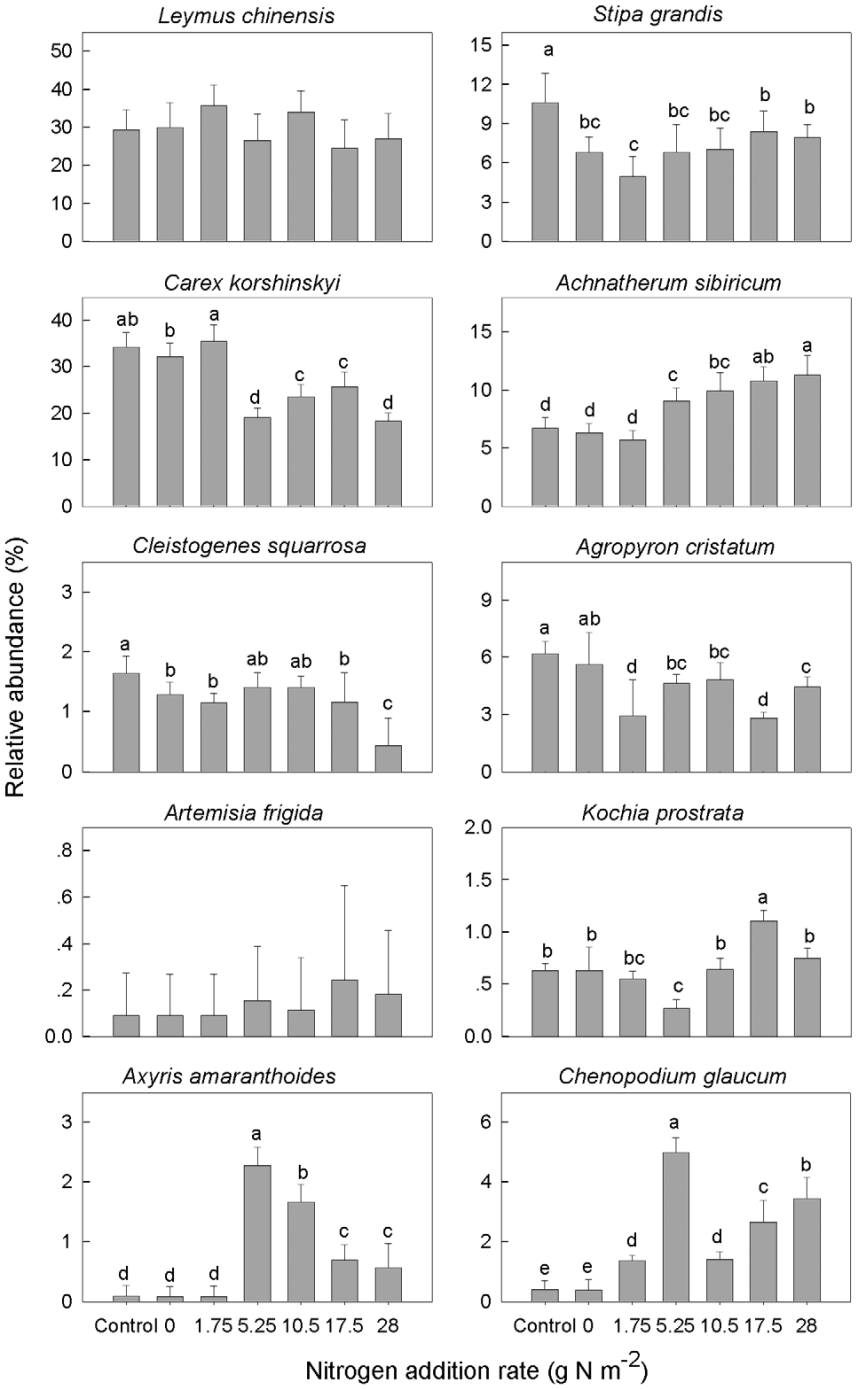
Relative abundance of each species in response to nitrogen addition. Bars are means ± s.d. Different letters between treatments indicate statistically significant differences determined by Duncan's multiple comparison.

Linear mixed model analysis indicated that species relative abundance was significantly affected by species, N addition rates, and their interaction (Table 1). The relative abundance of co-occurring species exhibited different response patterns to increasing N addition rates (Fig. 2). With increasing N addition rates, the relative abundance of bunchgrass species (*Stipa grandis*, *Cleistogenes squarrosa* and *Agropyron cristatum*) and the sedge species (*Carex korshinskyi*) decreased, while that of two annuals (*Axyris amaranthoides* and *Chenopodium glaucum*) and *Achnatherum sibiricum* increased. No significant change in relative abundance was observed for *Leymus chinensis*. Changes for the two semi-shrubs (*Artemisia frigida* and *Kochia prostrata*) did not show any consistent trend.

**Table 1:** F and *P* values of analysis of variance for the effects of nitrogen, species and their interaction (nitrogen×species) on aboveground biomass (AB), density, individual biomass (IB), and the ratio of aboveground allocation (AA) to belowground allocation (BA).

Fixed factors	d. f.	AB		Density		IB		AA/BA	
		F	*P*	F	*P*	F	*P*	F	*P*
Nitrogen	6	71.18	<.0001	70.19	<.0001	192.21	<.0001	104.12	<.0001
Species	9	878.75	<.0001	2554.31	<.0001	906.56	<.0001	1346.29	<.0001
Nitrogen×Species	54	58.13	<.0001	45.9	<.0001	55.75	<.0001	5.05	<.0001

### Individual biomass

**Figure 3:**
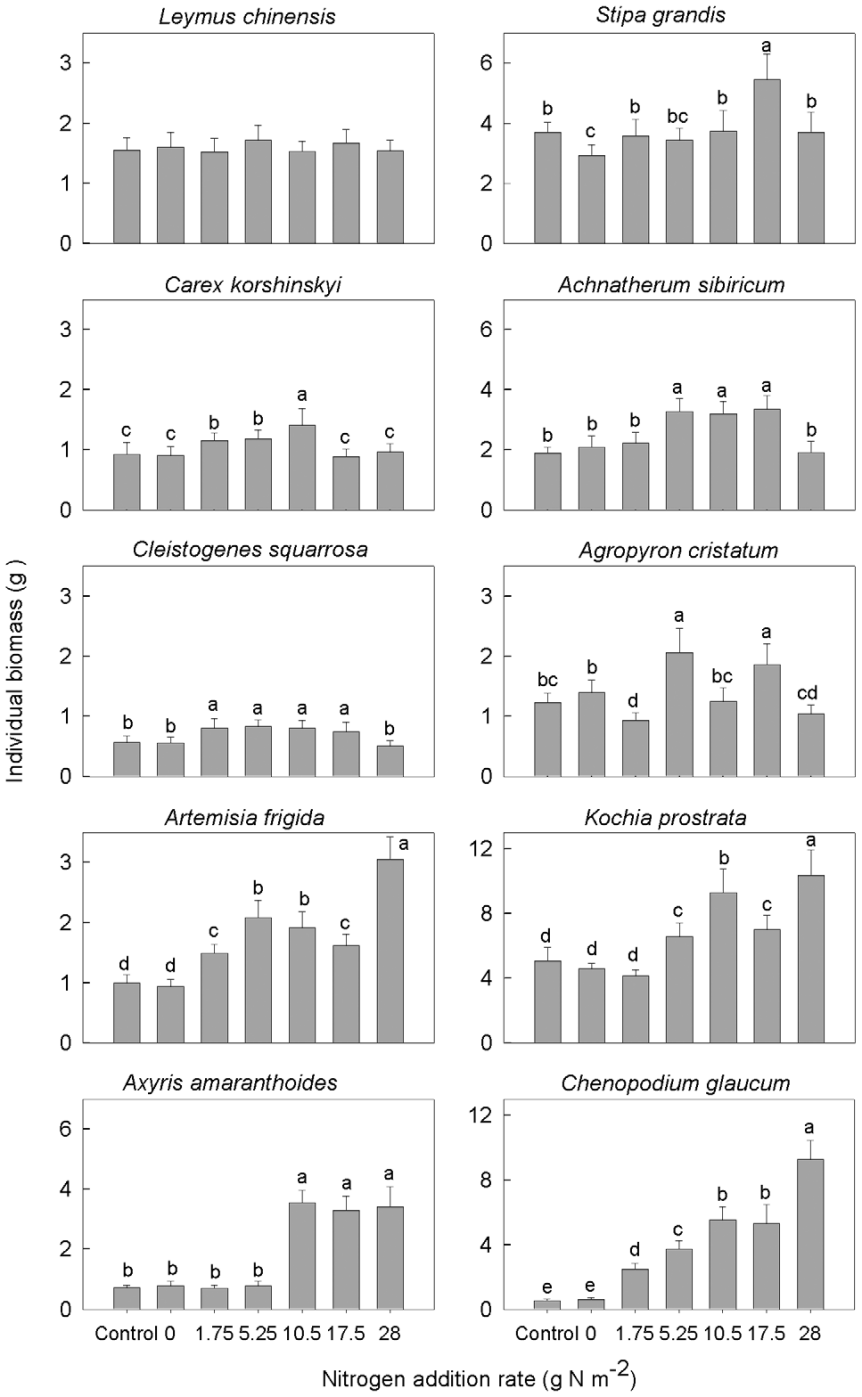
Individual biomass of each species in response to nitrogen addition. Bars are means ± s.d. Different letters between treatments indicate statistically significant differences determined by Duncan's multiple comparison.

The biomass of individual plants was significantly correlated with species, N addition rates, and their interaction (Table 1). The ten target species differed significantly in individual biomass response thresholds and the overall response patterns (Fig. 3). Four species (*C. korshinskyi*, *C. squarrosa*, *A. frigida*, *C. glaucum*) displayed a threshold at 1.75 g m^−2^ y^−1^, three species (*A. sibiricum*, *A. cristatum*, *K. prostrate*) at 5.25 g m^−2^ y^−1^, *Axyris amaranthoides* at 10.5 g m^−2^ y^−1^, and *S. grandis* at 17.5 g m^−2^ y^−1^. *L. chinensis* did not show any significant change. With increasing N addition rates, the individual biomass of four bunchgrasses and the *Sedge* species exhibited a hump-shaped response pattern, whereas two semi-shrubs and *C. glaucum* showed a linear response pattern.

To determine the differences between species and the plasticity within a species, the means and CVs of individual biomass for the ten species were analyzed (Table 2). *K. prostrata* had the highest mean individual biomass while *C. squarrosa* the lowest. Annuals exhibited the highest CVs, followed by semi-shrubs, whereas grasses and forbs exhibited the lowest CVs. Differences in individual biomass between species were larger than plasticity within species.

**Table 2:** Means and CVs of plant traits at the individual level (individual biomass, aboveground allocation, and belowground allocation).

Species	Individual biomass		Aboveground allocation		Belowground allocation	
	Mean (g)	CV (%)	Mean (%)	CV (%)	Mean (%)	CV (%)
*Leymus chinensis*	1.59d	13.58	21.59h	17.96	78.41a	4.95
*Stipa grandis*	3.79b	24.14	25.94g	16.05	74.06b	5.62
*Carex korshinskyi*	1.06ef	23.18	25.68g	18.42	74.32b	6.36
*Achnatherum sibiricum*	2.56c	28.34	30.33e	22.01	69.67d	9.58
*Cleistogenes squarrosa*	0.69f	25.59	27.02fg	21.84	72.98bc	8.08
*Agropyron cristatum*	1.40de	32.72	28.96ef	21.03	71.04cd	8.57
*Artemisia frigida*	1.73d	41.18	51.57d	12.59	48.43e	13.40
*Kochia prostrata*	6.72a	35.91	56.15c	14.42	43.85f	18.46
*Axyris amaranthoides*	1.90d	72.68	65.38b	12.70	34.62g	23.99
*Chenopodium glaucum*	3.92b	75.85	69.34a	6.73	30.66h	15.23
*Among species*		85.42		45.17		30.36

Numbers with the same letter are not statistically different at the significance level of 0.05.

### Biomass allocation

**Figure 4:**
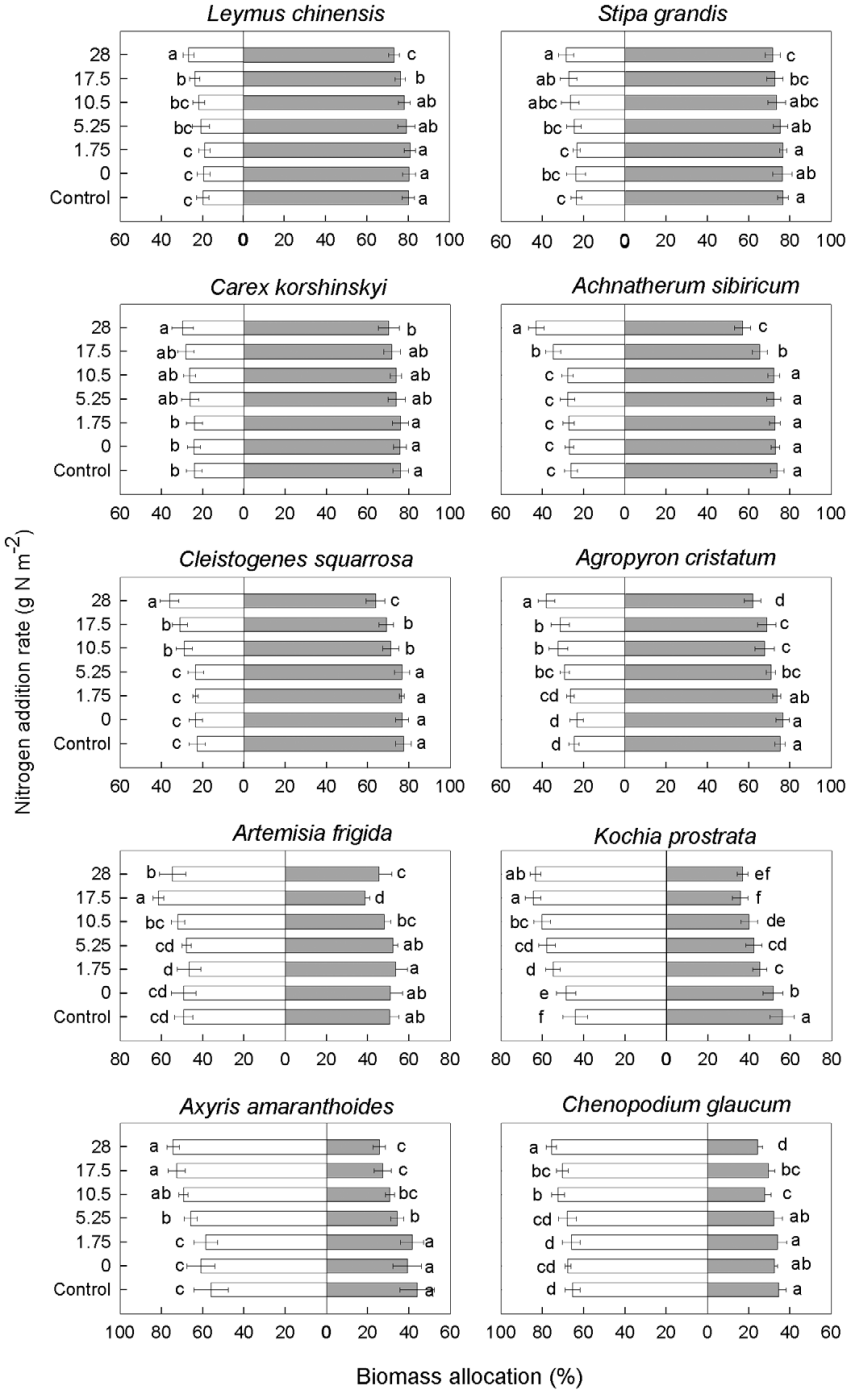
Biomass allocation of each species in response to nitrogen addition. Unfilled bars represent aboveground allocations and solid bars represent belowground allocations. Bars are means ± s.d. Different letters between treatments indicate statistically significant differences determined by Duncan's multiple comparison.

Overall, biomass allocation was also significantly correlated with species, N addition rates, and their interaction (Table 1). Following N addition, all species increased aboveground allocation and decreased belowground allocation, although their response patterns differed significantly (Fig. 4). The grasses and one forb species had higher mean belowground allocation with lower belowground plasticity, while the semi-shrubs and annuals showed higher mean aboveground allocation with lower aboveground plasticity (Table 2). Between-species differences in allocation pattern were larger than within-species plasticity.

### Plant responses to nitrogen addition rates at different organizational levels

**Figure 5:**
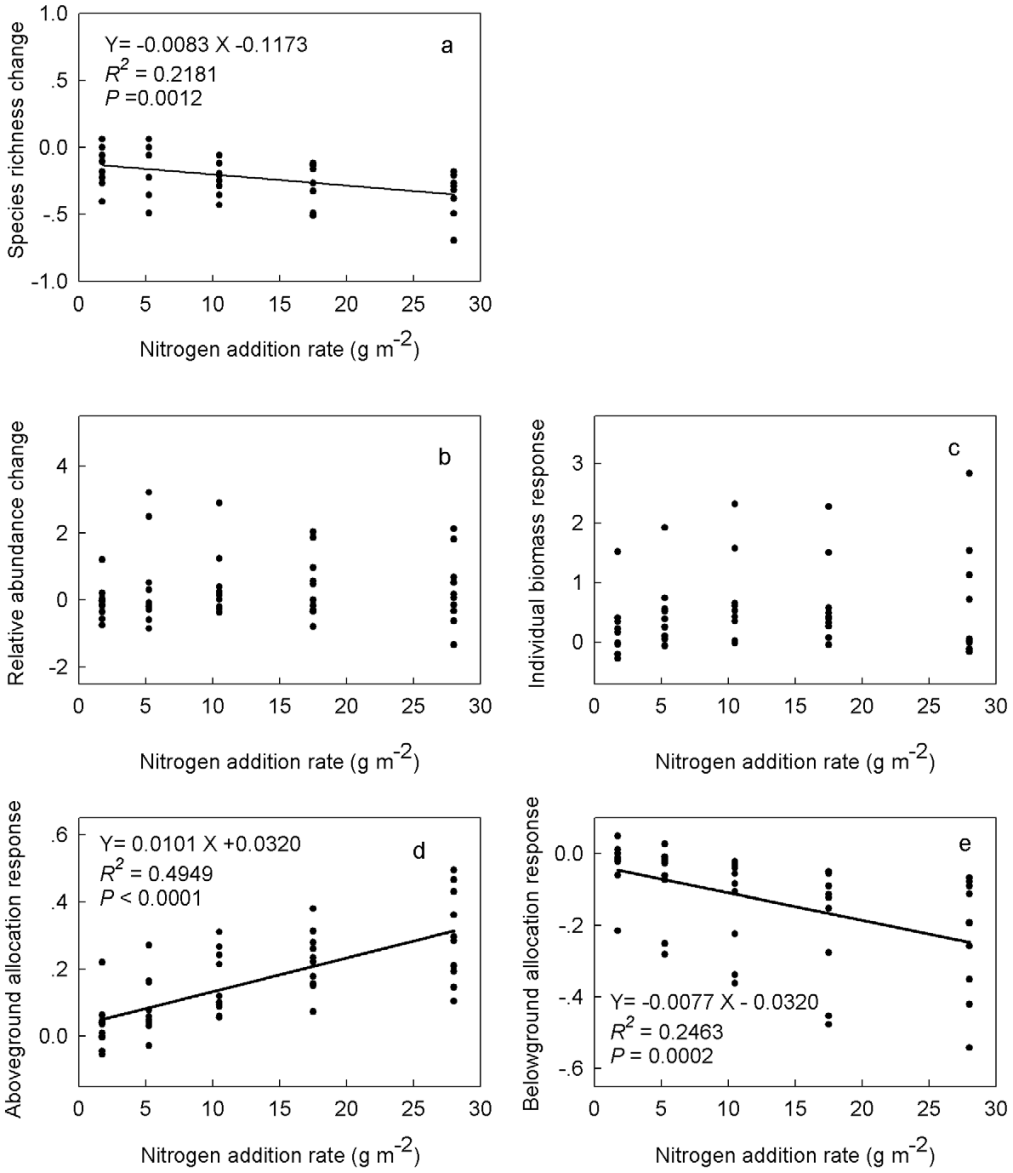
Relationships of species richness changes (a), species relative abundance changes (b), individual biomass responses (c), aboveground allocation responses (d), and belowground allocation responses (e) to increasing nitrogen addition rates.

At the community level, species richness was negatively correlated with N addition rates (Fig. 5a). At the species level, no significant relationship was found between the variability in species relative abundance (V_RAB_) and N addition rates (Fig. 5b). At the individual level, no relationship was found between individual biomass responses and N addition rates (Fig. 5c). The response of aboveground allocation (R_AA_) was positively correlated with N addition rates (Fig. 5d), and the response of belowground allocation (R_BA_) was negatively correlated with N addition rates (Fig. 5e).

**Figure 6:**
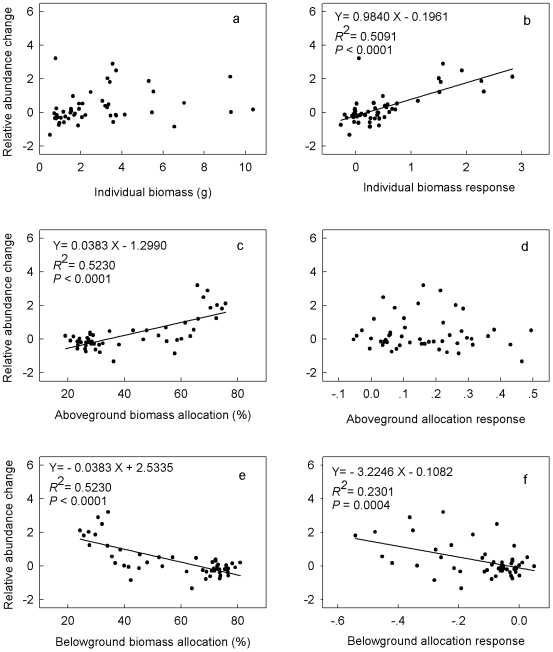
Relationships of species relative abundance changes to individual biomass (a), individual biomass responses (b), aboveground allocation pattern (c), aboveground allocation responses (d), belowground allocation pattern (e), and belowground allocation responses (f).

While the species-level changes in relative abundance (V_RAB_) were not significantly correlated with individual biomass (Fig. 6a), they were significantly positively correlated with individual biomass responses (R_IB_, Fig. 6b) and aboveground allocation (Fig. 6c). In addition, changes in species relative abundance were negatively correlated with belowground allocation (Fig. 6d) and belowground allocation responses (Fig. 6f).

## Discussion

### Hierarchical linkages between plant responses to N addition

N addition directly changes the biotic and abiotic environmental factors, which in turn acts on co-occurring plants [19], [28], [29]. For a given species, its overall response is the product of the responses of all individuals. In turn, all species-level responses together determine the community-level changes. Surprisingly, little is known about plant responses at different organizational levels and their hierarchical linkages. In the current study, we examined plant responses to two-year N addition and the linkages between these responses across three organizational levels. Our results indicated that co-occurring plants differed significantly in their responses to N addition across organizational levels. At the community level, as most N addition experiments have shown, species richness declined with increasing N addition rates [30], [31]. At the species level, however, no consistent relationship was observed between species relative abundance and N addition rates. This is mainly due to the differential responses of co-occurring species in terms of both the direction and magnitude of changes. Some species decreased their relative abundance, some increased their relative abundance, and others did not respond. At the individual level, a strong relationship was found between the responses of biomass allocation and N addition rates. These findings together provide a more comprehensive understanding of plant responses to N addition at the levels of individuals, species, and the entire community.

Our results also suggest that there are hierarchical linkages between the responses at different organizational levels, and the changes at higher organizational levels can be explained by the responses at lower organizational levels. First, the divergent responses at the species level provide a biological basis for explaining plant diversity loss at the community level. Specifically, our study indicates that the decline of perennial bunchgrasses and forbs, which dominate the Eurasian grasslands, was the dominant reason for the observed community-level species loss after N addition. Second, changes in species relative abundance were closely related to responses of individual biomass and belowground allocation. Moreover, we found that species-level changes were positively correlated with aboveground allocation pattern but negatively with belowground allocation pattern. These results suggest that both ecological functions and evolutionary strategies of species have important effects on species-level responses to N addition.

### Effects of allocation pattern and plasticity on plant responses

Biomass allocation pattern represents the evolutionary strategy of plant species for growth, reproduction, resource capture, and defense [18], [19], [32]. Tilman (1988) suggested that differences in allocation pattern may determine the order of species replacement after nutrient enrichment [19]. However, Olff (1992) argued that such between-species differences are not important because within-species plasticity in allocation pattern is much larger [24]. It is worth noting that Olff's (1992) conclusion was based on pot experiments with single species growing alone, which may not be relevant to the plasticity of plant species living together in a natural community. Our study clearly demonstrated that between-species differences, not within-species plasticity, in allocation pattern played a major role in determining plant responses to N addition. Considering that allocation plasticity may come from indirect effects of size-dependent allometric strategies [32], [33], the “true” plasticity from direct effects of N addition in our study system should be even smaller than our estimates. Thus, our findings support Tilman's prediction that differences in allocation pattern between species are key to the outcome of interspecific competition.

### Mechanisms of plant diversity loss due to N addition

Niu et al. (2008) reported that TCH was a major mechanism for species loss after N addition in an alpine meadow ecosystem [34]. However, our results were not consistent with the predictions of TCH because changes in above- and belowground allocations were not symmetric. The increased relative abundance of annuals and decreased relative abundance of perennial grasses together resulted in an overall increase in aboveground allocation at the community level because of the much higher aboveground allocation of annuals and the increased aboveground allocation responses for all species. There are at least two possible explanations for these contrasting results. First, Niu et al. (2008) only examined allocation patterns between different aboveground organs of plants, not directly between above- and belowground parts. Allocation strategies among plant aboveground parts may be different from those between above- and belowground parts. The second possibility is that the mechanisms of species loss after N addition may be system dependent. For example, most experiments in support of TCH were conducted in low-stature communities in which each species had at least some leaves exposed directly to sunlight [27]. The stature of plants in current ecosystem is much taller.

Our results did not support the predictions of BCH, either, because N addition favored species with low belowground allocation and because all species exhibited decreased belowground allocation responses. Also, changes in species relative abundance were negatively correlated with both belowground allocation patterns and belowground allocation responses, suggesting that belowground competition became less important after N addition. Our results were consistent with the predictions of ACH that competition for light is a major mechanism of plant species loss after N addition. First, N fertilization facilitated the growth of species with higher aboveground allocation (annuals) but suppressed the growth of those with higher belowground allocation (perennial grasses). After four years of N addition annuals became the dominant species in our study community [25]. Second, all species exhibited positive responses in aboveground allocation, and these responses were positively correlated with N addition rates, suggesting that aboveground competition was intensified after N addition. Third, changes in species relative abundance (V_RAB_) were positively correlated with aboveground allocation patterns, but negatively with belowground allocation patterns and responses. These results together strongly corroborate the predictions of aboveground competition hypothesis. Our finding is further supported by a light addition experiment that showed that species loss following N enrichment could be prevented by the addition of light to the grassland understory [35].

Although our results generally support ACH, some exceptions exist. For example, *A. sibiricum*, a perennial bunchgrass, displayed a strikingly different response pattern from other grasses with a similar allocation pattern. Also, two semi-shrubs had high aboveground allocation, but their relative abundance did not increase as did the two annuals. These exceptions suggest that factors other than allocation patterns, such as soil acidification [36] and litter accumulation [29], [37], may also play a role in influencing plant responses to N addition.

Although our results are based on a short-term experiment, the main conclusion that the increased aboveground competition is the primary mechanism for species loss following N addition in the Inner Mongolia grassland seems robust regardless of time scales as long as N enrichment continues. Our findings from this study have several implications for predicting the dynamics of plant diversity in grasslands with increasing N deposition and for the management practices of these ecosystems. In the Inner Mongolia grassland, perennial grasses are dominant species with well developed root systems that allow them to cope with environmental stresses, such as long-term drought and low N availability. N addition, however, ameliorates or even removes such limitation, thus disrupting the balance between above- and belowground allocations of resources. This disruption may lead to a decline of perennial grasses and forbs, and subsequently a shift in species dominance from perennial grasses to annuals. Such changes in community structure will inevitably result in alterations in ecosystem function and services, and reduce the resilience of these systems to climate change. Thus, controlling N enrichment from anthropogenic sources seems a necessary strategy for maintaining the biodiversity and ecosystem functioning of the Eurasian natural grasslands.

## Supporting Information

Table S1 Families and functional groups of ten examined species.
(DOC)
