# Supplementary material for: Hierarchical Plant Responses and Diversity Loss after Nitrogen Addition: Testing Three Functionally-Based Hypotheses in the Inner Mongolia Grassland
Source: PLoS One. 2011 May 19;6(5):e20078. doi: 10.1371/journal.pone.0020078 (PMC3098266; doi:10.1371/journal.pone.0020078)
Supplement: Table S1 — Families and functional groups of ten examined species. (DOC) [file pone.0020078.s001.doc]

***Table S1 Families and functional groups of ten examined species***

| Species | Family | Functional group |
| --- | --- | --- |
| *Leymus chinensis* | Gramineae | Perennial rhizome grass |
| *Stipa* *grandis* | Gramineae | Perennial bunchgrass |
| *Carex korshinskyi* | Cyperaceae | Perennial forb |
| *Achnatherum sibiricum* | Gramineae | Perennial bunchgrass |
| *Cleistogenes squarrosa* | Gramineae | Perennial bunchgrass |
| *Agropyron cristatum* | Gramineae | Perennial bunchgrass |
| *Artemisia frigida* | Compositae | Semi-shrub |
| *Kochia prostrata* | Chenopodiaceae | Semi-shrub |
| *Axyris amaranthoides* | Chenopodiaceae | Annual |
| *Chenopodium glaucum* | Chenopodiaceae | Annual |
